# Social media and deep learning capture the aesthetic quality of the landscape

**DOI:** 10.1038/s41598-021-99282-0

**Published:** 2021-10-08

**Authors:** Ilan Havinga, Diego Marcos, Patrick W. Bogaart, Lars Hein, Devis Tuia

**Affiliations:** 1 Environmental Systems Analysis Group, Wageningen University, Wageningen, 6708 PB The Netherlands; 2 Laboratory of Geo-Information Science and Remote Sensing, Wageningen University, Wageningen, 6708 PB The Netherlands; 3 National Accounts Department, Statistics Netherlands, The Hague, 2492 JP The Netherlands; 4 Environmental Computational Science and Earth Observation Laboratory, Ecole Polytechnique Fédérale de Lausanne, Industrie 17, Sion, Switzerland

**Keywords:** Sustainability, Environmental sciences

## Abstract

Peoples’ recreation and well-being are closely related to their aesthetic enjoyment of the landscape. Ecosystem service (ES) assessments record the aesthetic contributions of landscapes to peoples’ well-being in support of sustainable policy goals. However, the survey methods available to measure these contributions restrict modelling at large scales. As a result, most studies rely on environmental indicator models but these do not incorporate peoples’ actual use of the landscape. Now, social media has emerged as a rich new source of information to understand human-nature interactions while advances in deep learning have enabled large-scale analysis of the imagery uploaded to these platforms. In this study, we test the accuracy of Flickr and deep learning-based models of landscape quality using a crowdsourced survey in Great Britain. We find that this novel modelling approach generates a strong and comparable level of accuracy versus an indicator model and, in combination, captures additional aesthetic information. At the same time, social media provides a direct measure of individuals’ aesthetic enjoyment, a point of view inaccessible to indicator models, as well as a greater independence of the scale of measurement and insights into how peoples’ appreciation of the landscape changes over time. Our results show how social media and deep learning can support significant advances in modelling the aesthetic contributions of ecosystems for ES assessments.

## Introduction

Landscape aesthetics generate a large amount of cultural value for human well-being. The aesthetic quality of a landscape plays an important role in determining where people choose to recreate^1^. For example, recreational activities such as hiking are performed by people seeking aesthetic experiences related to the naturalness and perceived wilderness of a landscape^2^. As a consequence, the aesthetic contributions of ecosystems generated during peoples’ outdoor recreation are an important contributing factor to peoples’ mental and physical health^3^. The recent Covid-19 pandemic has especially highlighted the importance of outdoor recreation for peoples’ well-being^4,5^. Recreation is thus a key feature of environmental policy in Europe^6^. To capture this value and integrate it into land-use planning, ecosystem service (ES) models of recreation that consider the aesthetics of the landscape are being developed for use in European ES assessments^7^. ES assessments provide a science-policy interface through which the contributions of ecosystems to human well-being can be measured to achieve sustainable policy goals^8,9^.

Large-scale surveys can provide statistical measures of ES contributions based on peoples’ spatial interactions with the environment^10,11^. In the U.K., a recreational model was developed for the National Ecosystem Assessment using survey data on peoples’ outdoor recreation, the Monitor of Engagement with the Natural Environment (MENE). The model included land cover-based variables related to the aesthetic quality of the landscape^12^. However, due to their high cost and complexity, such large-scale surveys are rare. In this respect, the MENE survey in the U.K. is exceptional. Nevertheless, it only captures respondents’ spatial interactions based on a single gazetteer look-up, thereby missing finer-grained interactions that can tell us more about how and where people are benefiting from the landscape.

Due to these constraints, quantitative studies of aesthetic landscape quality are mostly based on spatially-explicit environmental indicators^1,13,14^. Common indicators include the presence of natural ecosystems, water, elevation, as well as spatial indices of landscape complexity such as the Patch Diversity Index (PDI) and the Shannon Diversity Index (SDI)^14–16^. The application of these indicators are based on visual concepts and theories developed in the landscape aesthetics literature^17,18^. However, crucially, these models do not incorporate peoples’ individual interactions with the environment, an important methodological factor from an ES modelling perspective^19–21^. Any measurements over time are also limited by updates to the underlying datasets which can take several years, an inflexible timeframe when considering the annual accounting requirements of some ES assessments^8^.

Recently, social media has emerged as a rich new source of information on human–nature interactions. The image-sharing platform Flickr has proven to be a particularly useful source of information. The locations of images and associated metadata, including tags and descriptions, have now been widely employed across the ES^22–26^, land use^27,28^ and landscape research literature^16,29,30^. Still, the data by themselves are difficult to interpret, mostly due to their volume and velocity. To respond to these challenges, researchers have turned to machine learning. In particular, deep learning, which uses artificial neural networks to generate predictions^31^. Supported by the increasing availability of training data and high-performance computer hardware, deep learning has made automatic image classification and object detection tasks possible over large datasets, including social media^32–35^. As a result, deep learning has been identified as an important new tool in the development of rapid, flexible and transferable cultural ES indicators^36^.

In the case of landscape aesthetics, an especially relevant training dataset exists: the Scenic-Or-Not (SoN) database. Through a web-based portal, the database has collected 1.5 million ‘scenicness’ ratings between 1 and 10 of 217,000 landscape images of Great Britain^37^. The images are sourced from Geograph, an online project to collect a geographically representative image of every square kilometre of the U.K. and Ireland. Studies have drawn on the SoN database to independently demonstrate both the potential of social media and machine learning in understanding peoples’ aesthetic preferences. Flickr metadata has been used to generate spatial predictions of scenic beauty^38^. Geograph tags have also been used to predict scenicness with random forests, a tree-based ensemble learning method for regression^39^. More recent studies have considered the image content directly using deep learning: image attributes related to the scenes and objects in SoN images have been used to generate scenicness predictions^40,41^. Subsequent research has focused on the detection of attribute groups co-influencing the perception of scenicness^42^, the discovery of new attributes using ancillary text corpora^43^, and the relationship between scenicness and land cover as observed by remote sensing satellites^44^.

These studies demonstrate the potential of modelling landscape aesthetics using social media and deep learning. At the same time, from an ES modelling perspective, social media provides the possibility of integrating peoples’ revealed preferences through their spatial interactions with the environment, and to observe the aesthetic contributions of landscapes with high spatial and temporal granularity^45^. This is in contrast to indicator-based models, which only take into account a general set of stated preferences, are limited by their spatial resolution and rely on updates to the underlying datasets to track temporal changes. Still, user activity on social media may not reflect common aesthetic preferences and could fail to detect significant changes over time. This is because studies validating the use of social media for cultural ES indicators are lacking^46^, a common problem in cultural ES studies^47^. Examining the accuracy of social media and deep learning in modelling landscape aesthetics versus an indicator-based approach will thus generate much-needed evidence confirming the potential benefits of using these novel techniques.

In this study, we compare models of landscape quality using Flickr and deep learning with an environmental indicator model, and explore their synergistic use. We generate spatial predictions for Great Britain using random forests and draw on the SoN database and its concept of scenicness to train and test our models. Flickr-based variables are generated using the predictions of two deep learning models at the image-level. The first, a pre-trained Places365-ResNet-50 model^48^, predicts scene classes and image attributes using the SUN database^49^. A scene class can be defined as the overall semantic description of an image while an image attribute is a specific characteristic within it (e.g. a collection of objects or human activity). The second model, a SoN ResNet, generates scenicness predictions in individual images. Environmental indicator variables are linked to visual concepts in the literature and are calculated using ecosystem type maps of Europe and other open-source data. We also analyse the effect of limiting Flickr user activity and examine aesthetic enjoyment over time in national park areas. Our findings illustrate how these innovative methods can advance ES modelling to achieve sustainable policy goals.

**Figure 1:**
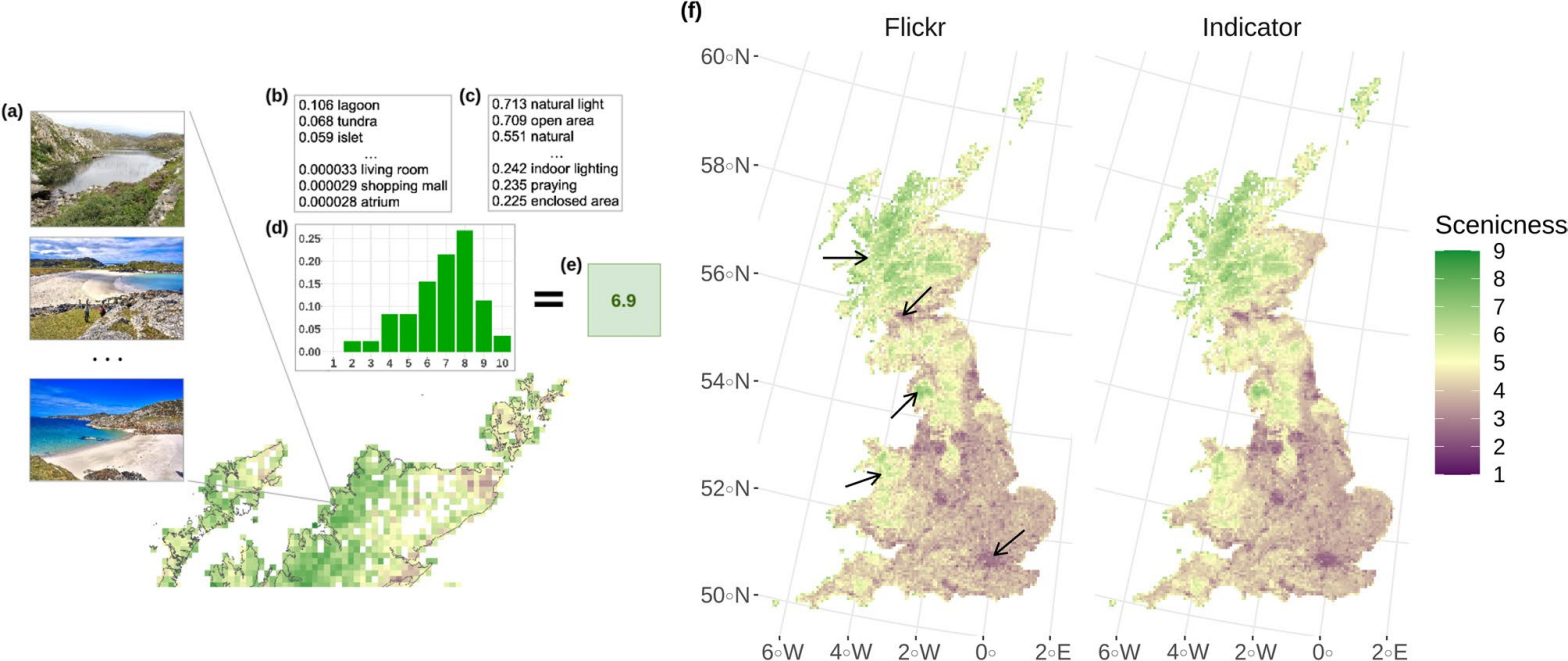
A Flickr and deep learning-based prediction of scenicness at 5 km resolution for a single cell covering Achmelvich Bay, Scotland, and for Great Britain, in comparison to the indicator model. The deep learning-based models used (**a**) Flickr images to generate (**b**) 365 scene class scores and (**c**) 102 SUN attribute scores, as well as (**d**), a normalised scenic rating distribution. These were then used to build a random forest model to generate (**e**) a scenicness prediction. In (**f**), predictions of the best-performing Flickr model for Great Britain are shown alongside the indicator model. From top to bottom, the arrows point to the Scottish Highlands, Glasgow, the Lake District, Snowdonia National Park and London. Drawn using R 3.6.3. (https://www.r-project.org/) with the ggplot2 3.3.5 (https://ggplot2.tidyverse.org) and cowplot 1.1.0 (https://cran.r-project.org/package=cowplot packages). Photos © Sergio and © Graeme Churchard (cc-by/2.0).

## Results

*Scenicness predictions using Flickr images and deep learning.* An example of a Flickr and deep learning-based prediction for a single 5 $\times$ 5 km grid cell is shown in Fig. 1. Individual Flickr images (Fig. 1a) are passed through the Places365-ResNet-50 model to generate a grid cell mean for 365 scene classes (Fig. 1b) and 102 SUN image attributes scores (Fig. 1c), while image scenicness scores generated by the SoN ResNet are used to produce a normalised rating distribution between 1 and 10 (Fig. 1d). The scene class and image attribute scores show that, on average, the Places365-ResNet-50 model scored the images in the grid cell the highest for the “lagoon”, “tundra” and “islet’ scenes, and the lowest for “atrium”, “shopping mall” and “living room”. In terms of attributes, the images were scored the highest for “natural light”, “open area” and “natural” while “enclosed area”, “praying” and “indoor lighting” received the lowest scores. A full list of image attribute and scene classes is available in Supplementary Tables S1 and S2 online. The normalised rating distribution shows that most images were rated 7 and above by the SoN ResNet. The predictions produced by the two deep learning models were then used as individual variables in a random forest model which predicted a final scenicness score of 6.9 for the grid cell (Fig. 1e).

**Table 1:** Scenicness model accuracy results on the gridded test set at 5 km resolution, derived from the SoN database.

Model	Places365 scene classes	SUN attributes	Scenic rating distribution	Environmental indicators	${r^2}$	RMSE	Kendall’s ${\tau }$
**Flickr**							
1	–	–	✓	–	0.659	0.639	0.611
2	–	✓	–	–	0.754	0.542	0.671
3	–	✓	✓	–	0.757	0.540	0.672
4	✓	–	–	–	0.757	0.541	0.677
5	✓	–	✓	–	0.757	0.541	0.677
6	✓	✓	✓	–	0.766	0.529	0.680
7	✓	✓	–	–	0.770	0.525	0.683
**Indicator**							
8	–	–	–	✓	0.819	0.468	0.730
**Combination**							
9	✓	✓	–	✓	0.827	0.458	0.732
10	✓	✓	✓	✓	0.827	0.457	0.733
11	–	✓	–	✓	0.830	**0.453**	0.734
12	✓	–	–	✓	**0.832**	0.453	0.738
13	–	–	✓	✓	0.830	0.453	**0.739**

**Figure 2:**
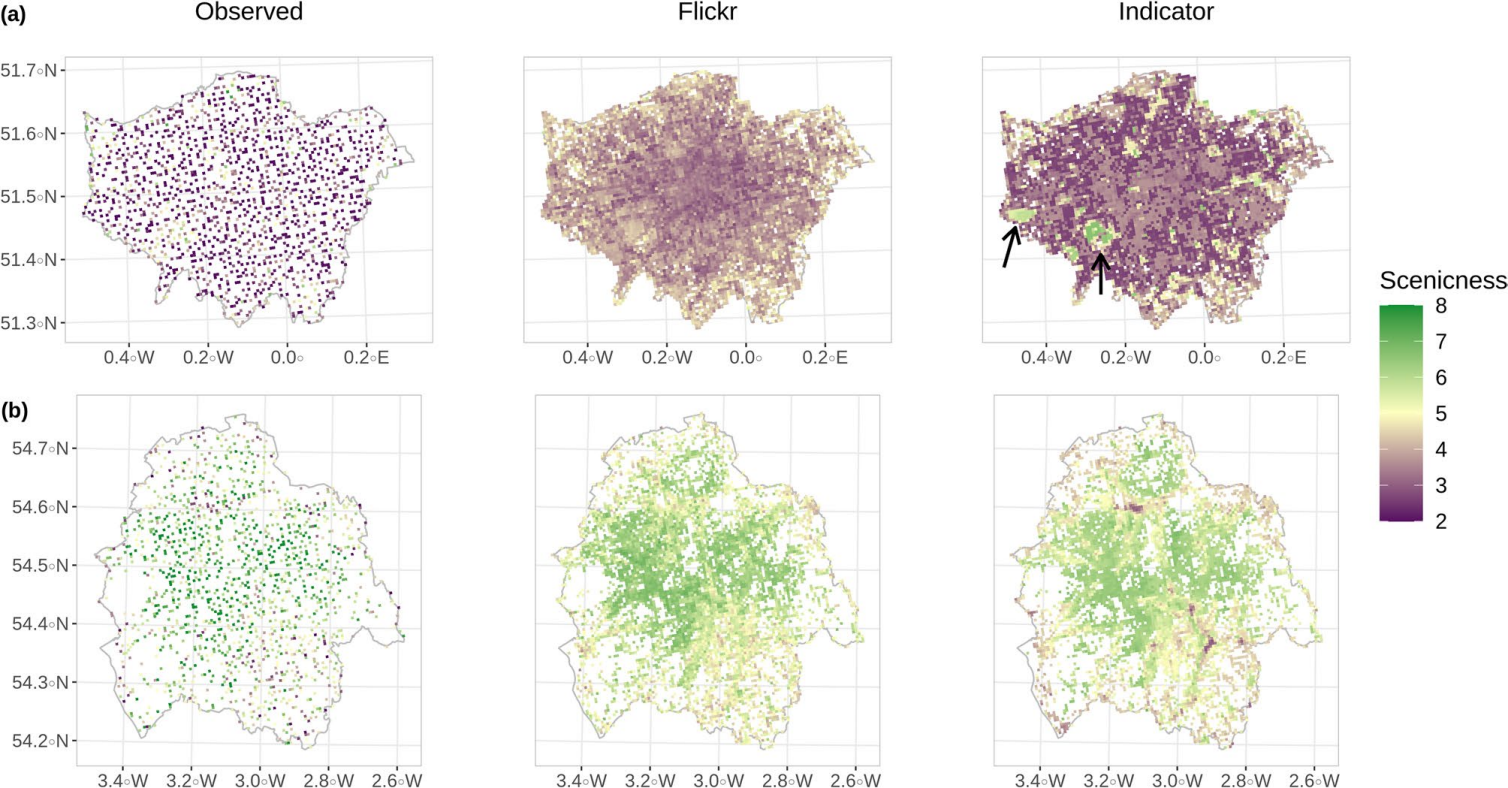
Observed scenicness versus the spatial predictions generated by the best-performing Flickr model and indicator model at 500 m resolution in (**a**) the Greater London area and (**b**) the Lake District national park. The arrows within the Greater London indicator model map point to Heathrow Airport (left) and Richmond Park (right). The observed versus predicted grid cell values are shown in Supplementary Fig. S1 online. Drawn using R 3.6.3 (https://www.r-project.org/) with the ggplot2 3.3.5 (https://ggplot2.tidyverse.org) and cowplot 1.1.0 (https://cran.r-project.org/package=cowplot) packages.

*Comparison of Flickr, environmental indicator and combined models.* The accuracy of the random forest models using the Flickr and deep learning-based variables, environmental indicators, and different combinations of the two, within a 20% hold-out test area are show in Table 1. Accuracy is reported using $r^2$, root mean squared error (RMSE) and Kendall’s $\tau$, a ranking correlation coefficient between − 1 (*inverse correlation*) and 1 (*absolute correlation*). Using Kendall’s $\tau$ to rank the models, the best-performing Flickr model used the Places365 scene classes and SUN attributes as variables. The model achieved a $\tau$ of 0.683 versus 0.730 achieved by the indicator model. Model performance was maximised when the environmental indicator variables and the scenic rating distribution were combined, producing a $\tau$ of 0.739.

**Figure 3:**
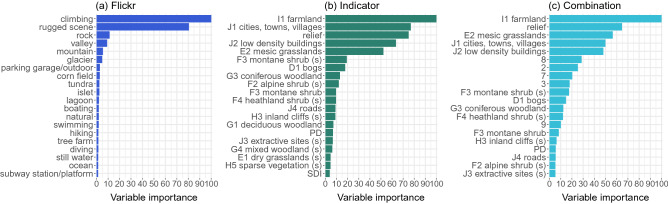
Most important variables for (**a**) the best-performing Flickr model, (**b**) environmental indicator model, and (**c**) best-performing combination model at 5 km resolution. “(s)” denotes an ecosystem in surrounding area variable. Supplementary Table S3 online contains a full list of ecosystem type codes and class descriptions. Drawn using R 3.6.3 (https://www.r-project.org/) and the ggplot2 3.3.5 (https://ggplot2.tidyverse.org) package.

The spatial predictions generated by the best-performing Flickr model and indicator model for the whole of Great Britain at 5 km grid cell resolution are shown in Fig. 1f. The two model types produced very similar spatial predictions. Areas of particularly high aesthetic value are captured well by both models, such as Snowdonia National Park in Wales, the Lake District in England and the Scottish Highlands. Similarly, urban areas of less scenic quality such as London in England and Glasgow in Scotland, are also clearly visible. In Fig. 2, a more detailed comparison is shown of the model predictions at 500*m* resolution versus the observed values. In both the Greater London area (Fig. 2a) and in the Lake District (Fig. 2b), we see more nuanced predictions using the Flickr model, while the indicator model produces more extreme values and sharp boundaries. For example, in Greater London, Richmond Park and Heathrow Airport are predicted as very scenic areas in contrast to some of the neighbouring areas by the indicator model, while the predictions of the Flickr model are much more muted and in line with the observed values. In the Lake District, we also see more extreme values in the unscenic areas using the indicator model, while the Flickr model behaves again in a more conservative manner. Overall, the Flickr model predictions in both areas show more consistency with the observed values, although the least scenic areas in the Lake District are less visible.

Variable importance for the Flickr, environmental indicator and combination models at 5 km resolution are shown in Fig. 3. The best-performing Flickr model, which used the Places365 scene classes and SUN attributes as variables, mainly drew on “climbing” and “rugged scene” in making its predictions. Natural scenes and attributes closely related to landscape aesthetics were also prominent such as “valley”, “mountain” and “natural”, as well as other recreation-related attributes such as “hiking”. The indicator model relied heavily on the presence of arable land and market gardens (I1), relief, and the presence of buildings (J1 and J2) to generate a scenicness prediction. This was followed by the presence of natural ecosystems, including grasslands (E2), mires/bogs (D1), heathland (F3s, F4s and F3), and inland scree/bare surfaces (H3s). The complexity indices SDI and PDI did not constitute important variables. The best-performing combined model, incorporating the scenic rating distribution (model 13, Table 1), drew on a similar set of indicator variables and the more extreme scenic ratings, focusing on the distributions across rating bins 2, 3, 7 and 8.

**Figure 4:**
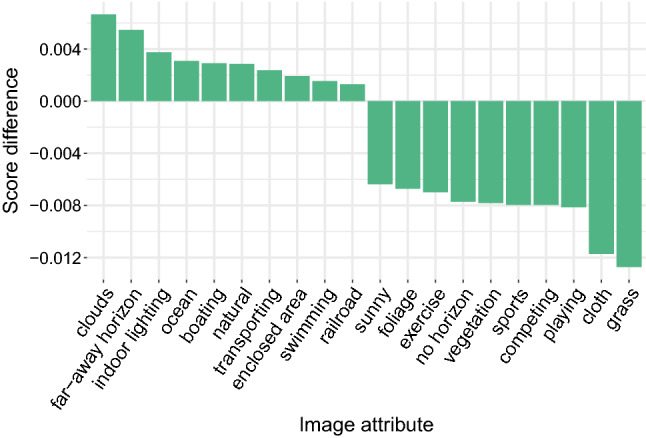
The largest differences in image attribute scores after limiting Flickr user contributions. To calculate the difference, a single image per user per day per grid cell was randomly selected ten times and the mean attribute scores were calculated per grid cell. The median difference versus the unfiltered dataset is shown here with summary statistics available in Supplementary Table S5 online. Drawn using R 3.6.3 (https://www.r-project.org/) and the ggplot2 3.3.5 (https://ggplot2.tidyverse.org) package.

*Limiting Flickr user activity.* For ES modelling purposes at national level, it is important to capture a representative measure of ecosystem contributions to human well-being. In the case of the Flickr models, accuracy results are reported after limiting individual Flickr users to one image per day per $5\times 5$ km grid cell. We applied the limitation after finding large geographic disparities in images per user (Supplementary Fig. S2 online). After applying the limitation, model accuracy improved versus a non-filtered dataset (Supplementary Table S4 online). Figure 4 shows the largest resulting change in image attribute confidence scores. A key change that can be observed is a decrease in the prevalence of images related to sporting. For example, “playing”, “competing”, “sports”, and “exercise” all saw notable decreases. This suggests that a large number of images associated with sporting events, less relevant for measuring landscape aesthetics, were removed from the dataset by the filtering. This in turn appears to have increased the prevalence of landscape-focused imagery, indicated by the increase in confidence scores for the “clouds”, “far-away horizon”, “ocean” and “natural” attributes.

**Figure 5:**
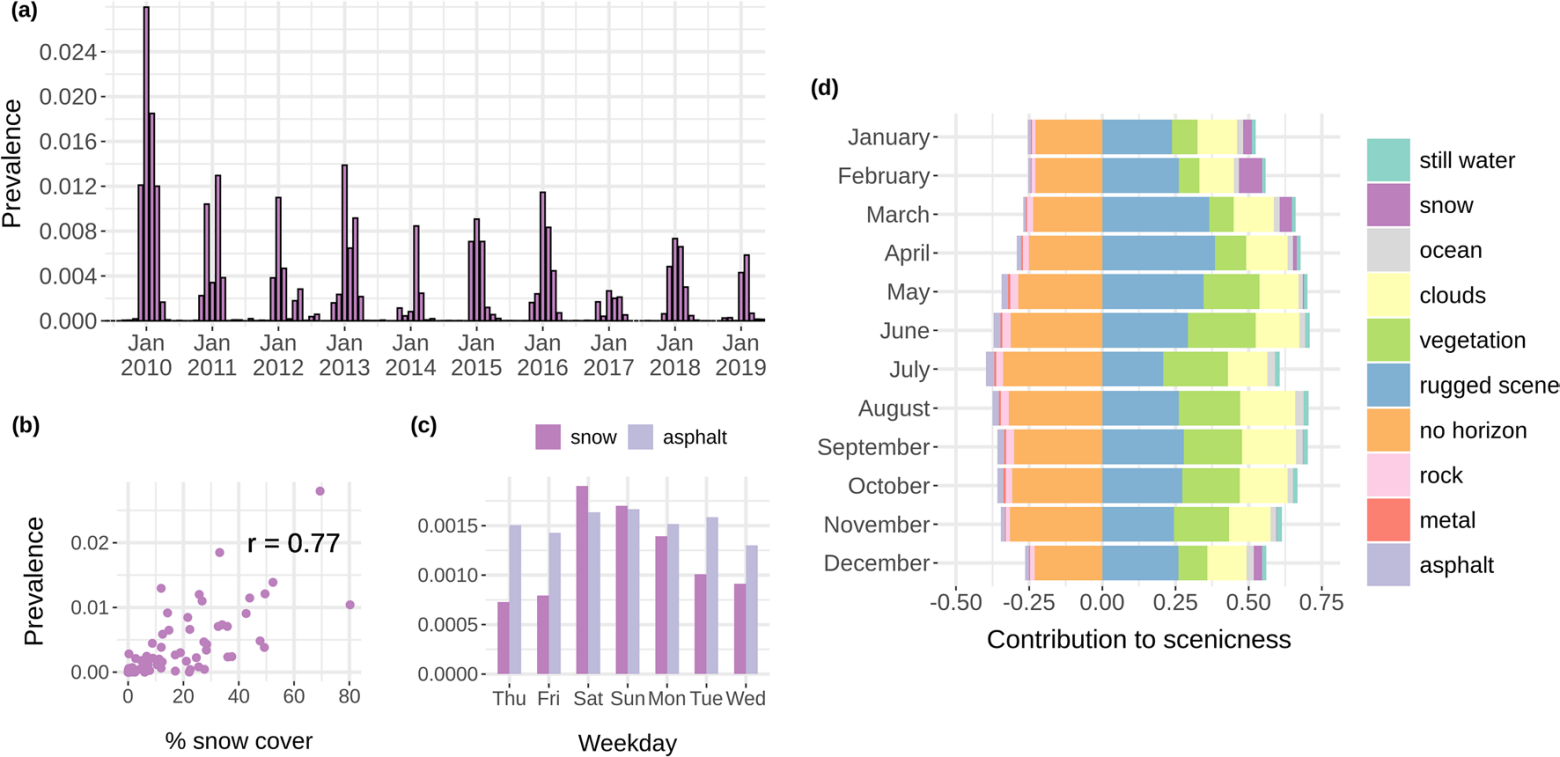
The influence of “snow” and other image attributes on aesthetic enjoyment over time. The (**a**) average monthly prevalence of “snow” attribute scores $>0.5$ are shown between 2009 and 2019. The average monthly scores show a strong correlation (Pearson’s $R=0.77$) with (**b**) remotely-sensed snow cover data. The (**c**) average prevalence per weekday of “snow” versus “asphalt” is also shown. A constrained linear model ($R^2=0.34$) was trained using the scores $>0.5$ of a selected group of attributes including “snow” to (**d**) predict scenicness at the image-level. The changing monthly contributions of some of these attributes can be observed, with “snow” contributing in the winter months. Contributions represent individual image attribute scores multiplied by the model coefficients and averaged on a monthly basis. Drawn using R 3.6.3 (https://www.r-project.org/) with the ggplot2 3.3.5 (https://ggplot2.tidyverse.org) and cowplot 1.1.0 (https://cran.r-project.org/package=cowplot) packages.

*Measuring changes in aesthetic enjoyment over time.* Deep learning-based variables generated using social media can also support measures of landscape aesthetics over time. This can support more frequent updates to national ES assessments, and tell us more about how the landscape is contributing to peoples’ well-being. In an additional experiment aiming at studying the temporal dynamics of peoples’ aesthetic enjoyment through their interactions with the landscape, we analysed how scenicness evolves over time in national park areas. Figure 5 shows the contributions of a selected group of image attributes over a ten year period within the 15 national parks of Great Britain. These contain some of the most valuable natural areas in Britain, such as the Peak and Lake Districts in England, the Pembrokeshire coast in Wales, and the Cairngorms in Scotland.

The contribution of aesthetic-related image attributes change in these national parks according to the season. We focus on the “snow” attribute as a specific example of how these contributions change over time. Figure 5a shows how the prevalence of “snow”, the average score accounting only for images with a score higher than 0.5, increases in the winter months. The winter of 2009/2010 reveals itself as a particularly snowy period. The prevalence of snow in user images correlates strongly with remote sensing-based measurements of snow cover using MODIS satellite data, shown in Fig. 5b. In Fig. 5c, we also see how the prevalence of “snow” increases around the weekend when people are more likely to visit snowy landscapes, whilst the prevalence of “asphalt” in images remains relatively constant throughout the week. This shows that the use of social media-based data provides a combination of information about the state of the environment and how people interact with it.

In a direct connection to aesthetic landscape quality, when the selected group of image attributes shown in Fig. 5d, including “snow”, are used to predict the image ratings generated by the SoN ResNet, we see again how the contributions change over time. For example, the contributions of “snow” appear between December and April, reaching a peak in the winter month of February, before disappearing again. In contrast, the contributions of “vegetation” grow to their highest between June and August, reflecting the positive influence of deciduous growth on landscape aesthetics in the summer. Although smaller in size, the contributions of “ocean” also grow in the summer, suggesting an increase in user posts of coastal images to Flickr in these warmer months. It is also notable that the contribution of “rugged scene” to scenicness increases in the rainy months of spring.

## Discussion

The potential of social media and deep learning to capture peoples’ interactions with the landscape has yet to be fully confirmed. In an ES context, social media provides a rich new source of data to capture the cultural contributions of ecosystems to human well-being but its use is rarely validated^46^. In the ES community, deep learning applications also remain limited and those that do exist tend to limit their analysis to using the objects detected in images as proxies for cultural ES^25,36,50^. We have demonstrated that deep learning-based variables which consider the overall semantic meaning of an image can accurately capture the aesthetic quality of the British landscape. Crucially, these techniques also incorporate peoples’ actual interactions with the environment, a key methodological requirement from an ES perspective.

Nevertheless, our study highlights the relevance of traditional environmental indicator models in capturing landscape quality in the absence of survey data. The visual concepts put forward in the landscape aesthetics literature serve well to capture the spatial variation in scenicness provided by the SoN database. The especially strong influence of unnatural, man-made environments on aesthetics is reflected in the high variable importance of arable land and buildings^51^. At the same time, the importance of highly valued and unique natural environments, such as bog and heathland ecosystems, as well as the importance of relief, are also accurately identified by the random forest model^52–54^. Surprisingly, the SDI and PDI, normally key indicators for measuring landscape aesthetics^55^ and relevant to Britain^56^, did not constitute important variables in our results. The variety of ecosystem type indicators and their interaction in the non-linear model space may have offered enough opportunities to capture landscape complexity^57^. Alternatively, visibility modelling of the landscape could produce a more accurate set of indicators^21,58,59^. Theoretically, these could capture more of the aesthetic quality of the landscape by providing a 3D perspective using the location of Flickr images. However, the challenge with visibility modelling at very large scales is the computational resources needed for the geo-spatial calculations^60^. For example, in our case, the sightlines from 9.8 million images would need to be calculated using a $25\times 25$ m Digital Elevation Model (DEM) for a 210,000 ${\mathrm{km}}^2$ area. On the other hand, in the case of our Flickr model, the presence of image attributes including “far-away horizon” and scene classes such as “mountain” give the model a lot of indirect information on the 3D characteristics of an area.

The inclusion of individual spatial interactions offered by the Flickr and deep learning-based approach also makes it a more attractive method for ES modelling purposes. The comparable model accuracy versus the indicator model shows that this key methodological requirement from an ES perspective can be incorporated without significant losses in accuracy. The results also show that this individual perspective produces a finer-grained view which captures highly-valued and unique landscape elements such as rock or water features^18^. For example, the highly aesthetic view of Achmelvich Bay in Scotland, shown in Fig. 1. This is in contrast to the indicator model, which uses variables measured with remote sensing data at 25 m resolution and above. At the same time, important negative environmental contexts, such as Heathrow Airport in London (Fig. 2), are also better captured by the Flickr model. Figure 2 also shows how the Flickr model stays relevant at different scales while simultaneously highlighting the scaling issues common to indicator models^61^. While the indicator model is heavily constrained by the scale of measurement, producing more extreme differences linked to land cover, the Flickr model is able to reproduce a more consistent view of the landscape using the images available to it (see also Supplementary Fig. S1 online). At a national level, it appears that explicitly capturing this more nuanced view of the landscape through the scenic rating distribution, in combination with the strong overall predictive power of the indicator model, produces the highest level of model accuracy in our study.

In contrast to the static nature of the indicator approach, the granularity of the Flickr data also enables a detailed examination of aesthetics over time. The time-series analysis illustrated in Fig. 5 shows how the aesthetic contributions of landscapes change over the course of a year in the national parks of Britain. The influence of seasonality on landscape quality, defined as ‘ephemera’ in the landscape aesthetics literature^17^, is notably captured. Such granularity can greatly benefit ES assessments requiring regular updates, such as those performed for the purposes of ecosystem accounting in the context of national annual accounts of economic production^8^. These results also show how the contributions of specific landscape characteristics to peoples’ aesthetic enjoyment can be accurately captured using a social media and deep learning-based approach. The large prevalence of snow in images during the 2009/2010 winter is consistent with one of the last great snowfall events in Britain^62^. The consistency with remote sensing data further supports the reliability of the data. Understanding how ecosystems in the landscape contribute to individuals’ aesthetic enjoyment of the landscape, and accurately tracking these contributions over time, can help policy-makers manage and protect the most valuable natural areas for peoples’ recreation and well-being.

Although the Flickr and deep learning approach has its advantages, some biases in the method should still be taken into account. By using the SoN database for training purposes, the models have largely learnt a British representation of aesthetic quality. For applications in other cultural and topographical contexts, additional fine-tuning will most likely be required. Challenges also lie in trying to gain an ES measure demographically-representative of the entire population. Flickr has been found to be the most popular with 40 to 60 year-old males^63^ and user contributions, as in our study, are usually skewed by small, highly active user groups^64^. At the same time, a great number of differences in the content of images exist and not all images are relevant for measuring landscape aesthetics. However, in this respect, the user limitation in our study appears to have shifted the overall image content away from sporting scenes and more towards landscape images, improving model accuracy versus the SoN database. Notably, the agreement between the Flickr-based models, SoN and the environmental indicators shows that there is a strong consistency between the preferences captured by each dataset. This consistency is also promising for applications in other European contexts as the aesthetic concepts used to develop the environmental indicators have already been successfully applied in a number of European settings^65^.

In conclusion, landscape aesthetics are an important source of cultural value but large-scale measurement for ES assessments is difficult due to a lack of survey data. Now, social media offers the opportunity to measure the aesthetic contributions of ecosystems whilst integrating peoples’ actual interactions with the environment, and tracking changes over time. In this study, we have demonstrated that models using Flickr images and deep learning enable a highly accurate measure of aesthetic landscape quality, with independence of the scale of measurement. This supports ES measures based on the revealed preferences of individuals rather than a set of broad theoretical concepts. Small gains in accuracy are also achieved when an explicit, deep learning-based measure of aesthetics in the form of an image rating distribution is combined with environmental indicator variables. Changes in the aesthetic contributions of landscapes over time can also be measured. Our results advance ES modelling to better capture the cultural contributions of nature to human well-being.

## Methods

**Figure 6:**
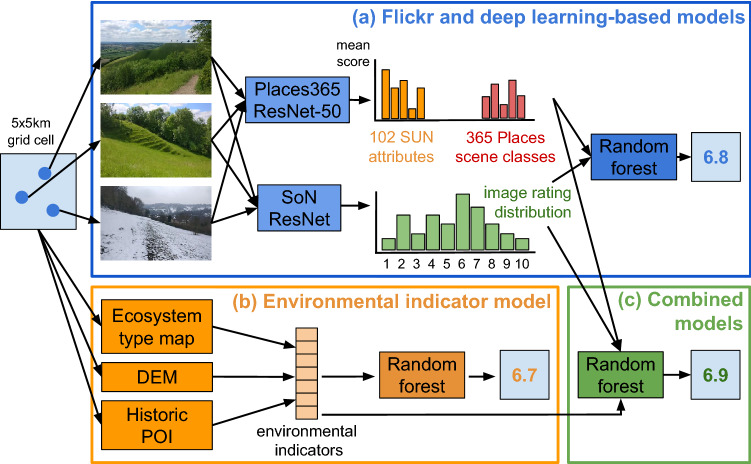
The main study design comparing spatial predictions of scenicness at a 5 $\times$ 5 km grid cell resolution using random forests. These used different combinations of variables based on (**a**) the predictions of two deep learning models on Flickr images, (**b**) a set of environmental indicators, and (**c**) combinations of the two. Photos © Alun Ward (cc-by/2.0).

*Study design.* The research focused on comparing Flickr and deep learning-based models with an environmental indicator-based model, as well as different combinations of the two (Fig. 6). Conceptually, we considered the aesthetic quality of the landscape equivalent to the concept of scenicness, and that scenicness constituted an integral factor determining the overall flow of aesthetic ES^66^. We made our comparisons using a $5\times 5$ km grid covering the entire terrestrial area of Great Britain and at 500 m resolution in Greater London and the Lake District. As a ground truth, we calculated a mean scenicness rating per grid cell using the image scenicness ratings of intersecting SoN images. Each image has a collection of volunteer ratings between 1 (not scenic) and 10 (very scenic). We used the average of these ratings. For training, we used the 5 km $\times$ 5 km grid. To reduce spatial autocorrelation, a larger 50 $\times$ 50 km grid was then overlaid onto this grid to create sample groups of which 70% was randomly allocated for training, 10% for validation and 20% for testing (Supplementary Fig. S3 online). Random forests was used to model scenicness at the 5 km and 500 m grid level using both the environmental indicator and Flickr-based variables. All spatial analyses were done using the R 3.6.3 programming language (https://www.r-project.org/) including the raster 3.0–12 (https://cran.r-project.org/package=raster), sf 1.0-1 (https://cran.r-project.org/package=sf), caret 6.0–86 (https://cran.r-project.org/package=caret) and tidyverse 1.3.1 (https://cran.r-project.org/package=tidyverse) packages. caret was used to automatically select the random forest hyperparameter settings *mtry*, *min node size*, and *extratrees*.

*Datasets.* In addition to the SoN database, a Flickr image dataset was compiled to generate the deep learning-based variables. To do this, image metadata for geo-located images taken in Great Britain between 2004 and 2020 were downloaded using Flickr’s API and accessed using the Python programming language. A script was developed which iterated over a 1 $\times$ 1 km grid, requesting the metadata of the 4000 most recent, geo-located images per grid cell. Geo-location accuracy was set to “street level”, the highest possible accuracy available through the API. The Places365-ResNet-50 model^48^ was then used to filter the dataset for outdoor images using its binary indoor/outdoor scene predictions. This resulted in a final dataset of 9.8 million outdoor images, resized to 250 $\times$ 250 pixel dimensions. The environmental indicator variables were also calculated using a number of geospatial datasets (Supplementary Table S6 online). These included the European Environment Agency (EEA) ecosystem type map, the EU DEM and OpenStreetMap. The EU GDPR on data protection and privacy were followed in the carrying out of the research.

*Flickr and deep learning-based variables.* To model scenicness at the grid level, spatial variables were generated using two deep learning models developed in Python 3.8.3 (Fig. 6). The pre-trained Places365-ResNet-50 model^48^ was applied to Flickr images to produce a first set of variables: a mean of 365 scene classes and 102 SUN image attribute scores per grid cell (a complete list is available in Supplementary Tables S1 and S2 online). Scene classes capture the overall semantic interpretation of an image, with scores representing probabilities between 0 and 1 based on the most likely scene out of 365 scene classes. Image attribute scores indicate the presence of objects and remarkable scene characteristics. These were normalised using a sigmoid function to produce a 0–1 probability per attribute. The second model, the SoN ResNet, was used to predict scenicness in Flickr images and to generate a second set of variables: a normalised count of its image predictions across ten scenic rating bins between 1 and 10, representing a scenic rating distribution per grid cell. We constructed this model using a modified ResNet-50 convolutional neural network, available pre-trained on the ImageNet database through the PyTorch 1.6.0 library. The final two layers of the network, originally designed to output confidence scores for ImageNet’s 1000 object classes, were removed and replaced with new layers designed to output an image scenicness score^42^. These consisted of an adaptive average pooling layer and two linear layers with a ReLU activation function on the output of the first linear layer. The network was trained and tested using SoN images according to the 70% training, 10% validation and 20% test areas. For training, this consisted of 152,470 images resized to $500\times 500$ pixel dimensions. Images were also randomly flipped horizontally to increase the size of the training dataset. Batch size was set to 16. Model weights were optimised using stochastic gradient descent and a mean squared error loss function. Test statistics are shown in Supplementary Table S7 online.

*Environmental indicator variables.* Variables were calculated per grid cell based on visual preference concepts put forward in the landscape aesthetics literature. The EEA ecosystem type map was used to calculate the percentage of different ecosystems to capture the naturalness of the landscape; relief in *m* was measured using the EU DEM to capture the aesthetic appeal of higher elevation areas and elevation differences; the PDI and SDI were calculated using the EEA ecosystem type map to measure landscape complexity; and, finally, to capture the uniqueness of natural environments and cultural elements in the landscape, the relative difference in the percentage area of ecosystems within 10 km was calculated, as well as the number of historical points of interest (POI) using OSM (Fig. 6). More details on the theoretical basis for these indicators and their calculation can be found in Supplementary Table S6 online.

*Environmental indicator reduction.* To improve model performance and interpretability, the initial environmental indicator set was reduced. First, ecosystem variables that could be calculated for less than $100 {\mathrm{km}}^2$ or $0.04\%$ of Great Britain were removed using a threshold analysis (Supplementary Fig. S4 online). Then, a check for collinearity between the remaining variables was performed. The model accuracy effect of removing variables with a correlation $r\ge 0.7$ was measured through a leave-one-out process in which random forest models were iteratively generated without one of the indicator variables in the full indicator set. The collinear variable with the smallest effect on model accuracy was removed (Supplementary Table S8 and Fig. S5 online). This resulted in a final indicator set of 41 variables.

*Time-series analysis.* An additional experiment was conducted to examine landscape aesthetics over time in the 15 national parks of Great Britain (Supplementary Fig. S6 online). Flickr images within these areas were extracted for the time period June 2009 to May 2019. The image attribute scores were extracted using the Places365-ResNet-50 model and prevalence was calculated on an image-level basis by taking only attribute scores greater than 0.5, subtracting 0.5 and multiplying by 2. All other values were set to 0. The linear model was trained and tested using a random 80/20% sample of images. MODIS snow cover data used the MOD10CM product which reports monthly average snow cover in 0.05$^\circ$. The centroids of the intersecting 5 $\times$ 5 km grid cells with national parks were used to extract percentage snow cover on a monthly basis. Additional spatial data sources are given in Supplementary Table S9 online.

## Supplementary Information

Supplementary Information.

## Data Availability

The data used in this study are open-source and publicly available. Code and data associated with this study can be obtained at 10.5281/zenodo.5534028.
