# Supplementary material for: Social media and deep learning capture the aesthetic quality of the landscape
Source: Sci Rep. 2021 Oct 8;11:20000. doi: 10.1038/s41598-021-99282-0 (PMC8501120; doi:10.1038/s41598-021-99282-0)
Supplement: Supplementary file 1 — Supplementary Information. [file 41598_2021_99282_MOESM1_ESM.pdf]

Supplementary Information for

Social media and deep learning capture the aesthetic quality of the landscape

Ilan Havinga*, Diego Marcos, Patrick W. Bogaart, Lars Hein, and Devis Tuia

*Corresponding author. Email: ilan.havinga@wur.nl

This PDF file includes:

Figs. S1 to S6
Tables S1 to S10
SI References

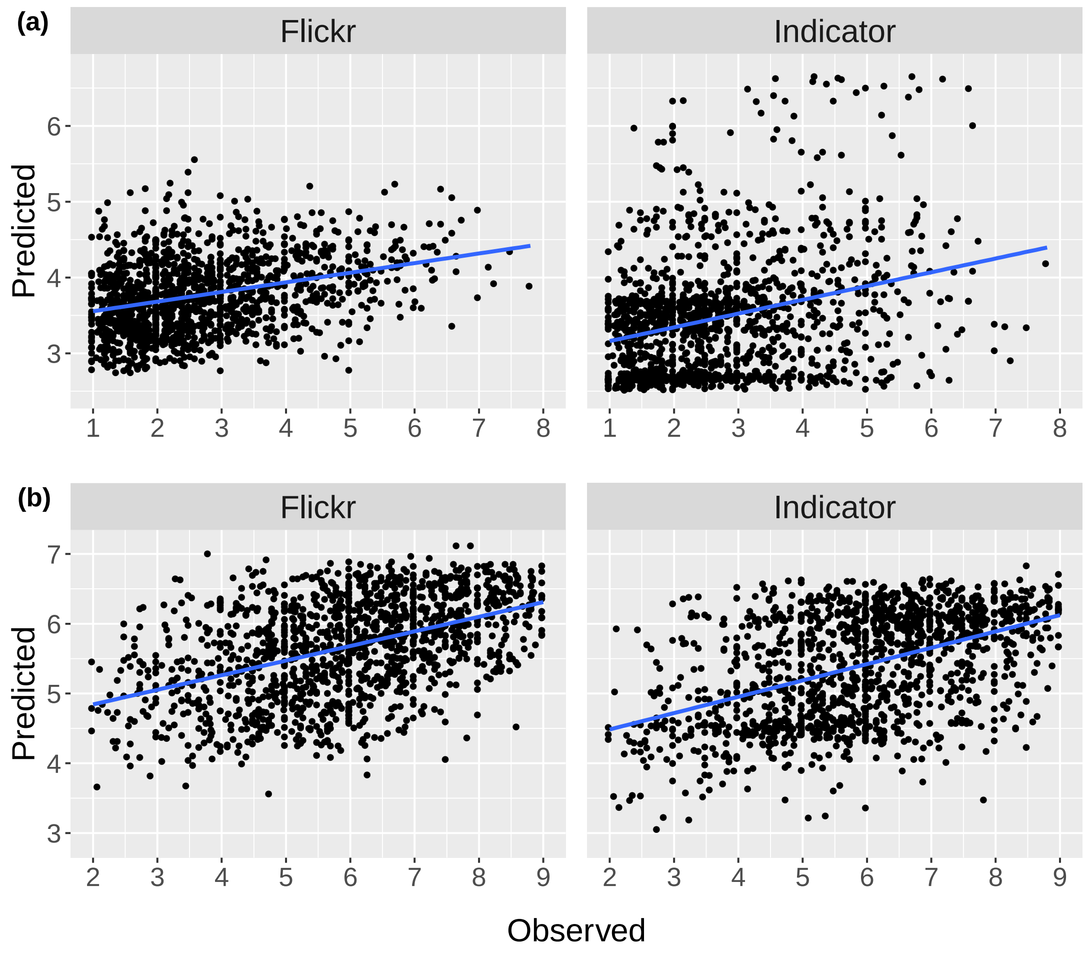

Fig. S1. Observed versus predicted values in (a) the Greater London area and (b) the Lake District. A Kendall's tau correlation test resulted in a 0.223 correlation for the Flickr model versus 0.175 for the indicator model in Greater London, and correlations of 0.334 versus 0.330 in the Lake District. Drawn using R 3.6.3 (https://www.r-project.org/) and the ggplot2 3.3.5 (https://ggplot2.tidyverse.org) package.

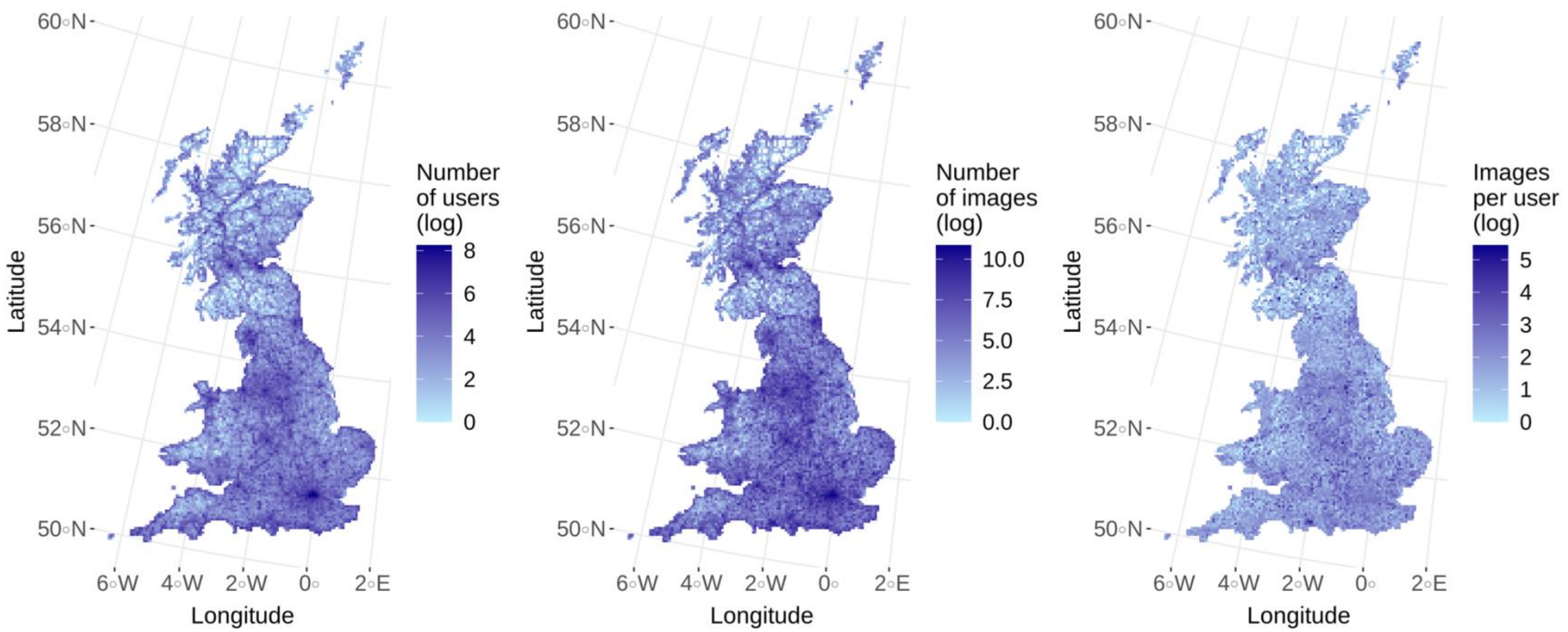

Fig. S2. Number of users, images and images per user in log scale. Drawn using R 3.6.3 (https://www.r-project.org/ with the ggplot2 3.3.5 (https://ggplot2.tidyverse.org) and gridExtra 2.3 (https://cran.r-project.org/package=gridextra) packages.

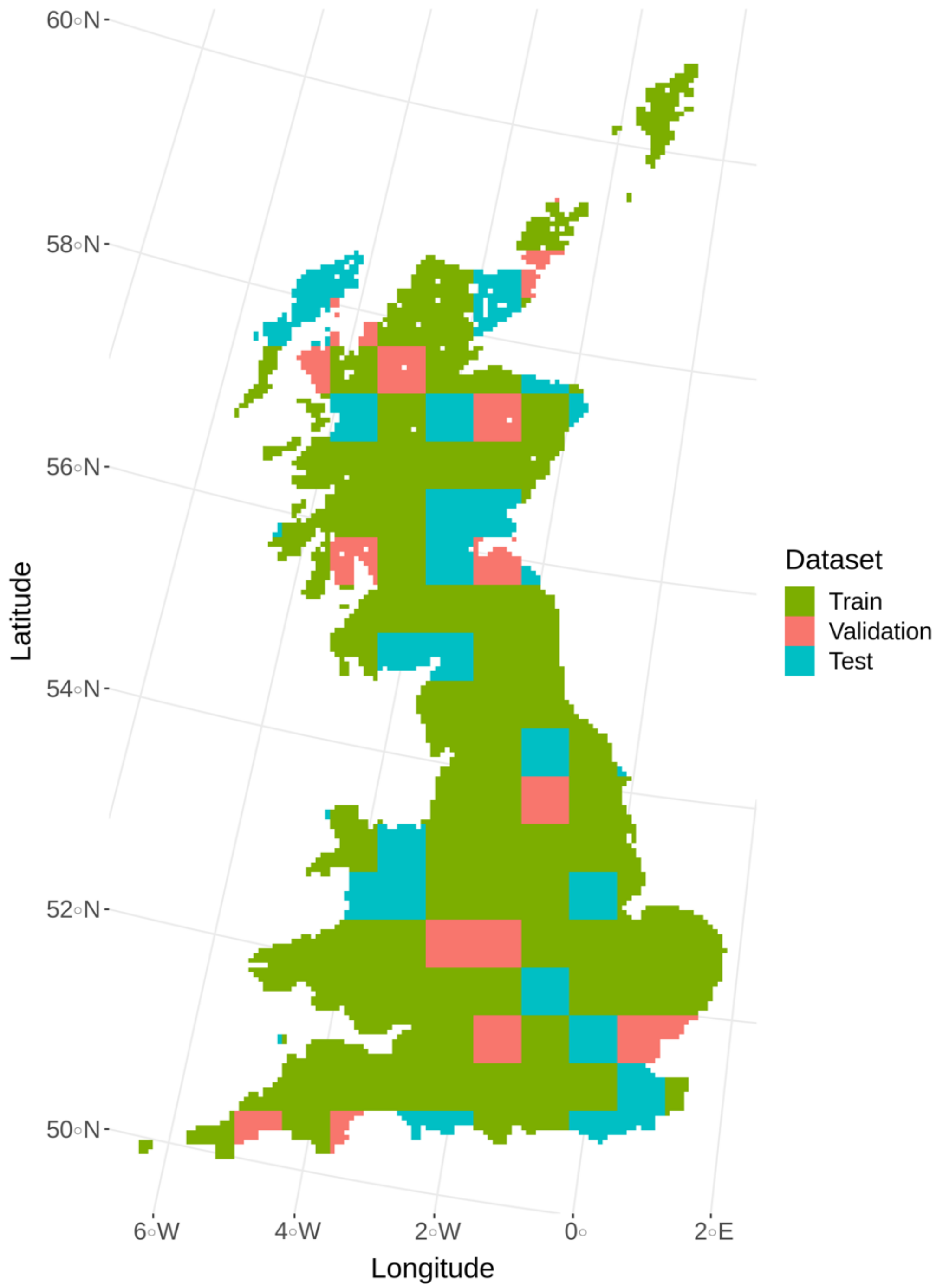

Fig. S3. The 70% training, 10% validation and and 20% test split used in the study. Drawn using R 3.6.3 (https://www.r-project.org/) and the ggplot2 3.3.5 (https://ggplot2.tidyverse.org) package.

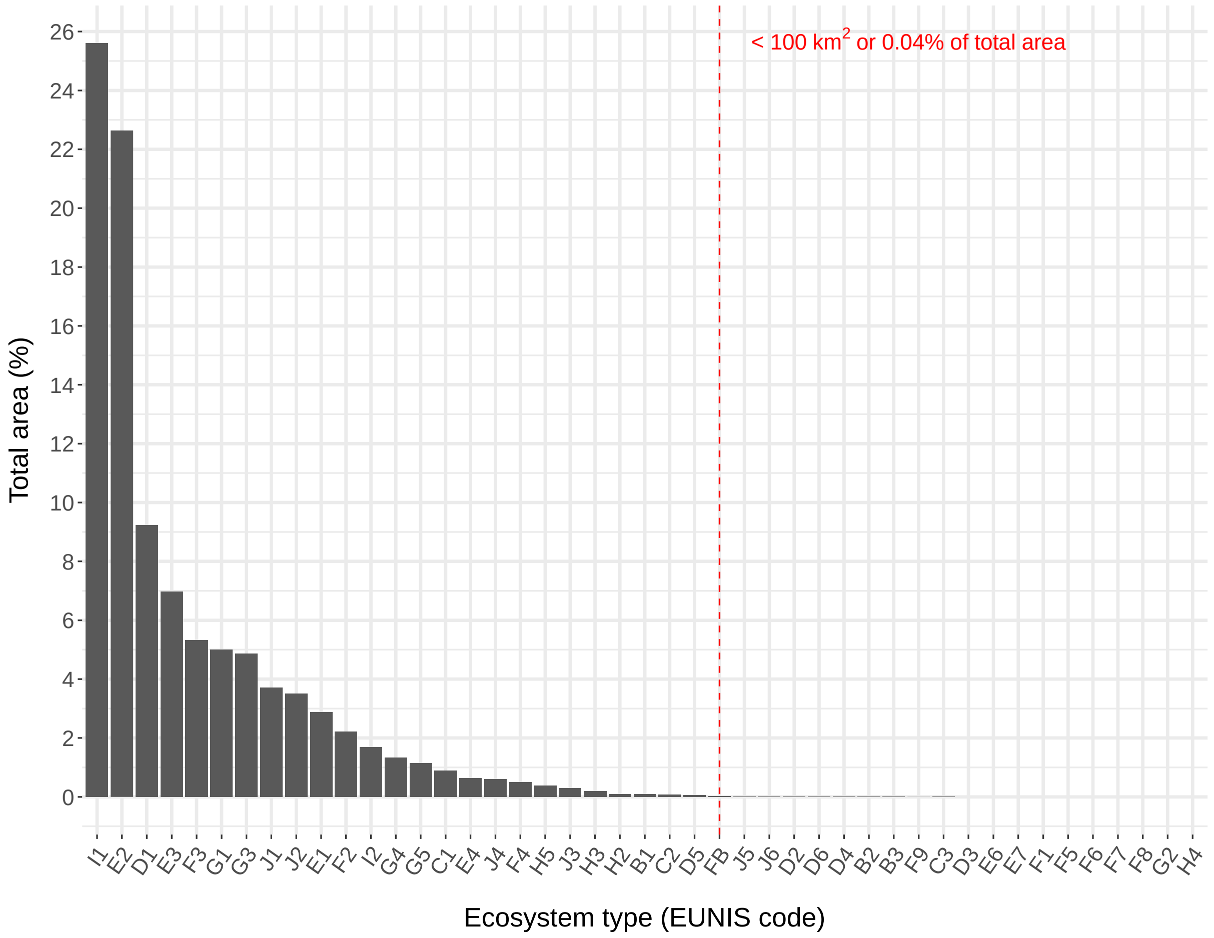

Fig. S4. Threshold analysis of ecosystem types in GB. B = coastal, C = water, D = mires/bogs, E = Grasslands, F = heathland, G = woodland, H = inland scree/bare surface, I = Arable land and market gardens, J = buildings. A threshold point at shrub plantations (FB) onwards was selected, after which very little or none of the ecosystem types are present in the country. Table S3 contains a full list of ecosystem code and class descriptions. Drawn using R 3.6.3 (https://www.r-project.org/) and the ggplot2 3.3.5 (https://ggplot2.tidyverse.org) package.

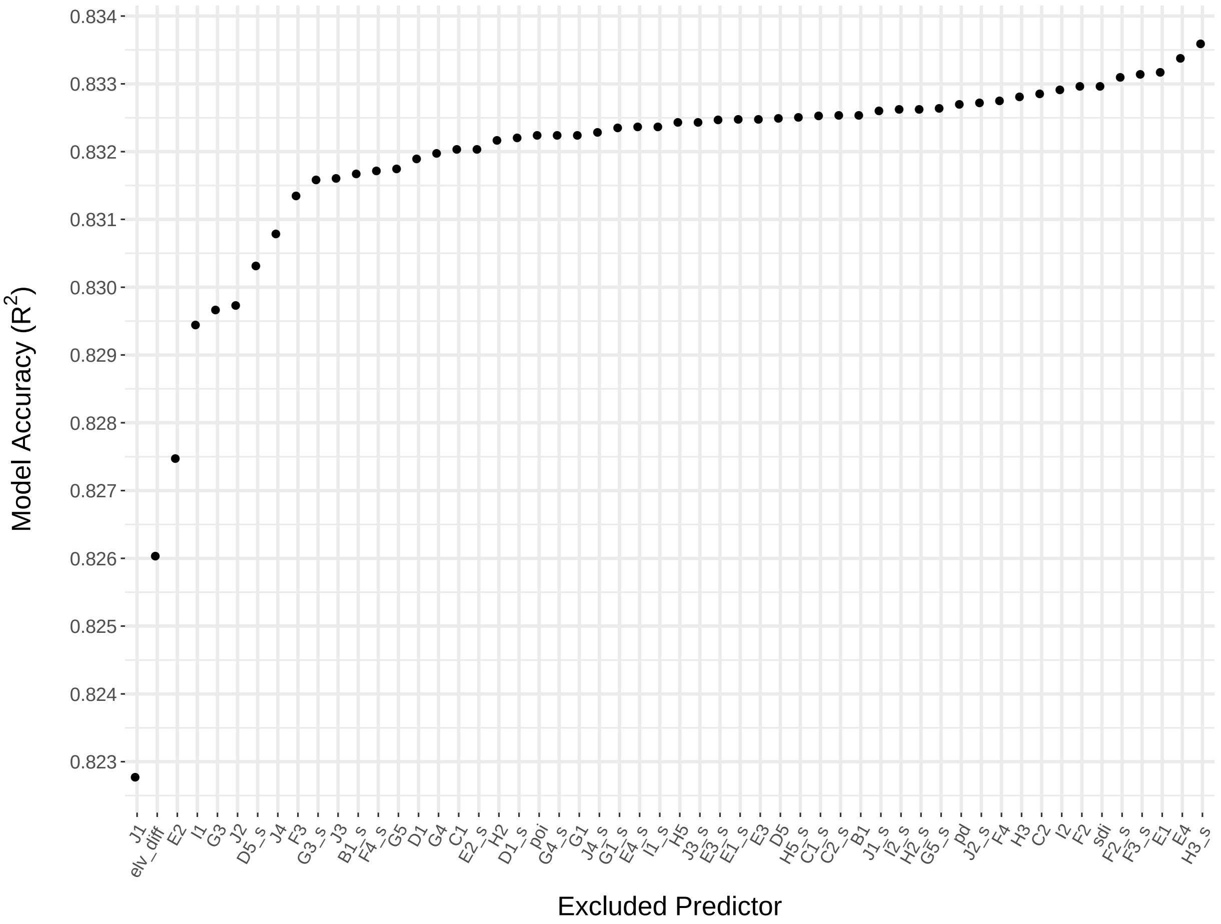

Fig. S5. Model accuracy (R^2) per excluded indicator. B = coastal, C = water, D = mires/bogs, E = Grasslands, elv_diff = relief, F = heathland, G = woodland, H = inland scree/bare surface, I = Arable land and market gardens, J = buildings. “s” denotes surrounding ecosystem indicator. Table S3 contains a full list of ecosystem code and class descriptions. Drawn using R 3.6.3 (https://www.r-project.org/) and the ggplot2 3.3.5 (https://ggplot2.tidyverse.org) package.

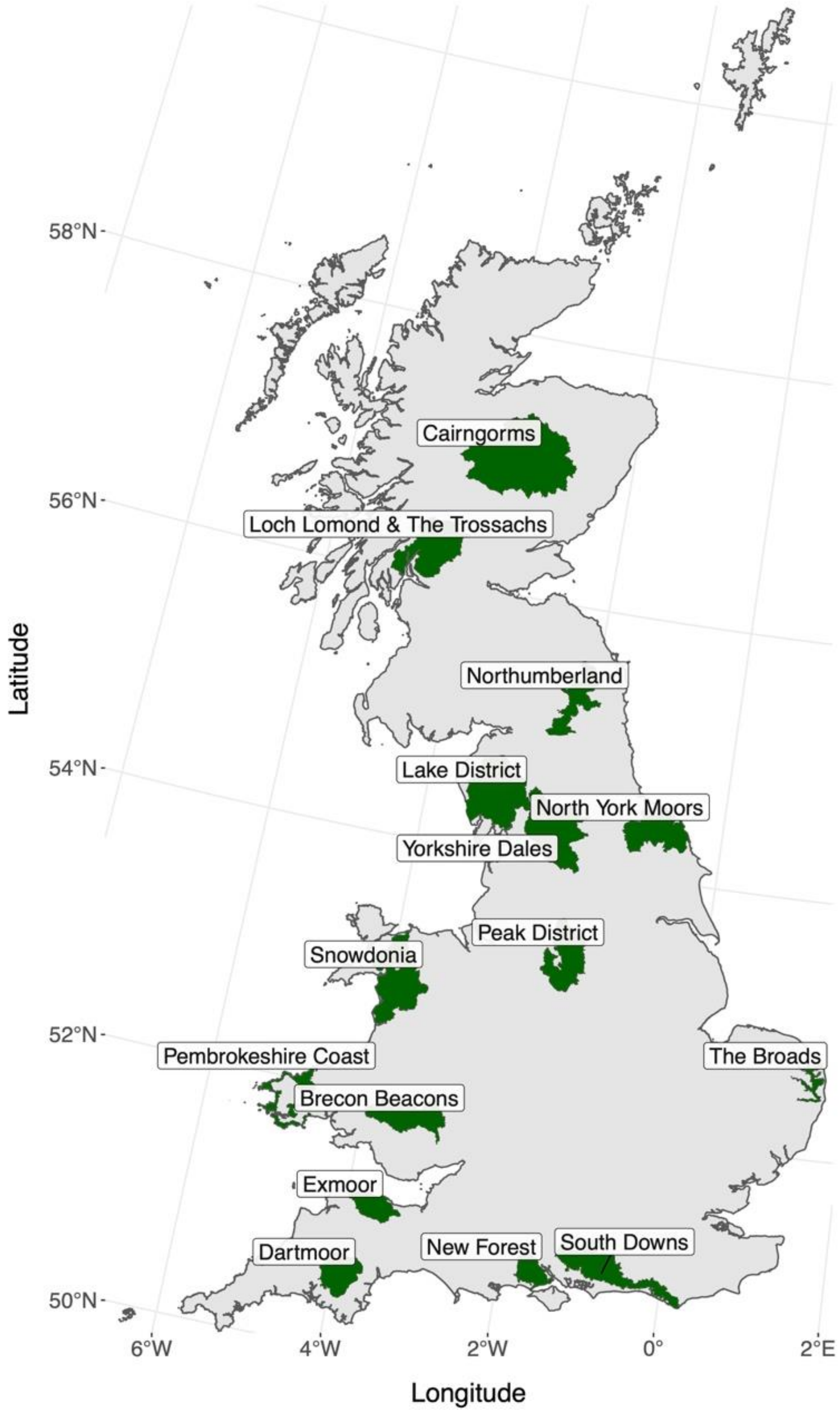

Fig. S6. National parks of Great Britain. Drawn using R 3.6.3 (https://www.r-project.org/) and the ggplot2 3.3.5 (https://ggplot2.tidyverse.org) package.

Table S1. Places365 scene classes.

airfield	bowling alley	drugstore	industrial area	parking garage / outdoor	subway station / platform
airplane cabin	boxing ring	elevator / door	inn / outdoor	parking lot	supermarket
airport terminal	bridge	elevator lobby	islet	pasture	sushi bar
alcove	building facade	elevator shaft	jacuzzi / indoor	patio	swamp
alley	bullring	embassy	jail cell	pavilion	swimming hole
amphitheater	burial chamber	engine room	japanese garden	pet shop	swimming pool / indoor
amusement arcade	bus interior	entrance hall	jewelry shop	pharmacy	swimming pool / outdoor
amusement park	bus station / indoor	escalator / indoor	junkyard	phone booth	synagogue / outdoor
apartment building / outdoor	butchers shop	excavation	kasbah	physics laboratory	television room
aquarium	butte	fabric store	kennel / outdoor	picnic area	television studio
aqueduct	cabin / outdoor	farm	kindergarden classroom	pier	temple / asia
arcade	cafeteria	fastfood restaurant	kitchen	pizzeria	throne room
arch	campsite	field / cultivated	lagoon	playground	ticket booth
archaelogical excavation	campus	field / wild	lake / natural	playroom	topiary garden
archive	canal / natural	field road	landfill	plaza	tower
arena / hockey	canal / urban	fire escape	landing deck	pond	toyshop
arena / performance	candy store	fire station	laundromat	porch	train interior
arena / rodeo	canyon	fishpond	lawn	promenade	train station / platform
army base	car interior	flea market / indoor	lecture room	pub / indoor	tree farm
art gallery	carrousel	florist shop / indoor	legislative chamber	racecourse	tree house
art school	castle	food court	library / indoor	raceway	trench
art studio	catacomb	football field	library / outdoor	raft	tundra
artists loft	cemetery	forest / broadleaf	lighthouse	railroad track	underwater / ocean deep
assembly line	chalet	forest path	living room	rainforest	utility room
athletic field / outdoor	chemistry lab	forest road	loading dock	reception	valley
atrium / public	childs room	formal garden	lobby	recreation room	vegetable garden
attic	church / indoor	fountain	Lock chamber	repair shop	veterinarians office
auditorium	church / outdoor	galley	locker room	residential neighborhood	viaduct
auto factory	classroom	garage / indoor	mansion	restaurant	village

auto showroom	clean room	garage / outdoor	manufactured home	restaurant kitchen	vineyard
badlands	cliff	gas station	market / indoor	restaurant patio	volcano
bakery / shop	closet	gazebo / exterior	market / outdoor	rice paddy	volleyball court / outdoor
balcony / exterior	clothing store	general store / indoor	marsh	river	waiting room
balcony / interior	coast	general store / outdoor	martial arts gym	rock arch	water park
ball pit	cockpit	gift shop	mausoleum	roof garden	water tower
ballroom	coffee shop	glacier	medina	rope bridge	waterfall
bamboo forest	computer room	golf course	mezzanine	ruin	watering hole
bank vault	conference center	greenhouse / indoor	moat / water	runway	wave
banquet hall	conference room	greenhouse / outdoor	mosque / outdoor	sandbox	wet bar
bar	construction site	grotto	motel	sauna	wheat field
barn	corn field	gymnasium / indoor	mountain	schoolhouse	wind farm
barndoor	corral	hangar / indoor	mountain path	science museum	windmill
baseball field	corridor	hangar / outdoor	mountain snowy	server room	yard
basement	cottage	harbor	movie theater / indoor	shed	youth hostel
basketball court / indoor	courthouse	hardware store	museum / indoor	shoe shop	zen garden
bathroom	courtyard	hayfield	museum / outdoor	shopfront	
bazaar / indoor	creek	heliport	music studio	shopping mall / indoor	
bazaar / outdoor	crevasse	highway	natural history museum	shower	
beach	crosswalk	home office	nursery	ski resort	
beach house	dam	home theater	nursing home	ski slope	
beauty salon	delicatessen	hospital	oast house	sky	
bedchamber	department store	hospital room	ocean	skyscraper	
bedroom	desert / sand	hot spring	office	slum	
beer garden	desert / vegetation	hotel / outdoor	office building	snowfield	
beer hall	desert road	hotel room	office cubicles	soccer field	
berth	diner / outdoor	house	oilrig	stable	
biology laboratory	dining hall	hunting lodge / outdoor	operating room	stadium / baseball	
boardwalk	dining room	ice cream parlor	orchard	stadium / football	
boat deck	discotheque	ice floe	orchestra pit	stadium / soccer	
boathouse	doorway / outdoor	ice shelf	pagoda	stage / indoor	

bookstore	dorm room	ice skating rink / indoor	palace	stage / outdoor	
booth / indoor	downtown	ice skating rink / outdoor	pantry	staircase	
botanical garden	dressing room	iceberg	park	storage room	
bow window / indoor	driveway	igloo	parking garage / indoor	street	

Table S2. SUN image attributes.

boating	competing	vegetation	marble	rusty
driving	sports	shrubbery	glass	warm
biking	exercise	foliage	surf	cold
transporting	playing	leaves	ocean	natural
sunbathing	gaming	flowers	running water	man-made
touring	spectating	asphalt	still water	open area
hiking	farming	pavement	ice	semi-enclosed area
climbing	constructing	shingles	snow	enclosed area
camping	shopping	carpet	clouds	far-away horizon
reading	medical activity	brick	smoke	no horizon
studying	working	& tiles	fire	rugged scene
training	using tools	concrete	natural light	vertical components
research	digging	metal	sunny	horizontal components
diving	conducting business	paper	indoor lighting	symmetrical
swimming	praying	wood	aged	cluttered space
bathing	fencing	vinyl	glossy	scary
eating	railing	plastic	matte	soothing
cleaning	wire	cloth	sterile	stressful
socializing	railroad	sand	moist	
congregating	trees	rock	dry	
waiting in line	grass	dirt	dirty	

Table S3. EEA ecosystem types.

EUNIS code	EUNIS Class name	Area (km^2)	Area (%)
B1	Coastal dunes and sandy shores	205.05	0.09
B2	Coastal shingle	15.75	0.01
B3	Rock cliffs, ledges and shores, including the supralittoral	9.79	0.00
C1	Surface standing waters	2027.55	0.88
C2	Surface running waters	177.16	0.08
C3	Littoral zone of inland surface waterbodies	3.74	0.00
D1	Raised and blanket bogs	21249.21	9.24
D2	Valley mires, poor fens and transition mires	23.34	0.01
D3	Aapa, palsa and polygon mires	0	0.00
D4	Base-rich fens and calcareous spring mires	17.31	0.01
D5	Sedge and reedbeds, normally without free-standing water	114.05	0.05
D6	Inland saline and brackish marshes and reedbeds	18.44	0.01
E1	Dry grasslands	6630.9	2.88
E2	Mesic grasslands	52083.24	22.64
E3	Seasonally wet and wet grasslands	16040.37	6.97
E4	Alpine and subalpine grasslands	1465.75	0.64
E6	Inland salt steppes	0	0.00
E7	Sparsely wooded grasslands	0	0.00
F1	Tundra	0	0.00
F2	Arctic, alpine and subalpine shrub	5106.45	2.22
F3	Temperate and mediterranean-montane scrub	12265.09	5.33
F4	Temperate shrub heathland	1146.42	0.50
F5	Maquis, arborescent matorral and thermo-Mediterranean brushes	0	0.00
F6	Garrigue	0	0.00
F7	Spiny Mediterranean heaths (phrygana, hedgehog-heaths and related coastal cliff vegetation)	0	0.00
F8	Thermo-Atlantic xerophytic scrub	0	0.00
F9	Riverine and fen scrubs	7.65	0.00
FB	Shrub plantation	51.22	0.02
G1	Broadleaved deciduous woodland	11485.08	4.99
G2	Broadleaved evergreen woodland	0	0.00
G3	Coniferous woodland	11202.43	4.87
G4	Mixed deciduous and coniferous woodland	3047.2	1.32
G5	Lines of trees, small anthropogenic woodlands, recently felled woodland, early-stage woodland and coppice	2619.83	1.14

H2	Screes	219.63	0.10
H3	Inland cliffs, rock pavements and outcrops	430.22	0.19
H4	Snow or ice-dominated habitats	0	0.00
H5	Miscellaneous inland habitats with very sparse or no vegetation	889.47	0.39
I1	Arable land and market gardens	58901.8	25.61
I2	Cultivated areas of gardens and parks	3895.57	1.69
J1	Buildings of cities, towns and villages	8526.9	3.71
J2	Low density buildings	8052.28	3.50
J3	Extractive industrial sites	674.15	0.29
J4	Transport networks and other constructed hard-surfaced areas	1385.46	0.60
J5	Highly artificial man-made waters and associated structures	26.75	0.01
J6	Waste deposits	23.85	0.01
**Total**		**230039.1**	**100**

Table S4. Scenicness model accuracy results without the user limitation.

	Model	Places365 scene classes	SUN attributes	Scenic rating distribution	Environmental indicators	r^2	**RMSE**	**Kendall's τ**
Flickr	1			✓		0.672	0.627	0.613
	2		✓			0.739	0.558	0.654
	3		✓	✓		0.746	0.552	0.660
	4	✓				0.756	0.542	0.674
	5	✓	✓	✓		0.760	0.537	0.674
	6	✓		✓		0.757	0.540	0.674
	7	✓	✓			0.762	0.534	0.675
Indicator	8				✓	0.819	0.468	0.730
Combination	9	✓	✓		✓	0.827	0.458	0.731
	10	✓	✓	✓	✓	0.827	0.458	0.732
	11		✓		✓	0.829	0.455	0.732
	12	✓			✓	0.830	0.456	0.734
	13			✓	✓	**0.831**	**0.453**	**0.738**

Table S5. Summary statistics for largest differences in image attribute scores with unfiltered dataset based on ten random image selections per user per day per grid cell.

Attribute	min	0.25	median	0.75	max
grass	-0.0129345	-0.012813	-0.012728	-0.0126741	-0.0125904
cloth	-0.0117806	-0.0117536	-0.0117219	-0.0116848	-0.0116245
playing	-0.0082253	-0.0081799	-0.00814	-0.0081338	-0.0081115
competing	-0.0080414	-0.0079843	-0.0079614	-0.0079484	-0.0079092
sports	-0.0080318	-0.0079829	-0.0079552	-0.0079414	-0.0078951
vegetation	-0.0080157	-0.0078309	-0.0078205	-0.0077516	-0.0075968
no horizon	-0.0079613	-0.0078365	-0.0077178	-0.0076277	-0.0075967
exercise	-0.0070634	-0.0070209	-0.0069846	-0.0069786	-0.0069431
foliage	-0.0068866	-0.0067719	-0.0067143	-0.006672	-0.0064665
sunny	-0.0065354	-0.0064305	-0.0063678	-0.0063338	-0.0062988
railroad	0.00125414	0.00129332	0.00129991	0.00130359	0.00134532
swimming	0.00144828	0.00147526	0.00154538	0.001581	0.00161854
enclosed area	0.00188263	0.00191018	0.0019217	0.0019345	0.00200661
transporting	0.00230039	0.00233472	0.00236526	0.00239926	0.0024206
natural	0.00271528	0.00278886	0.00285209	0.00304828	0.00318441
boating	0.00280333	0.00284384	0.00291285	0.00296349	0.00302866
ocean	0.00295043	0.00301037	0.00308272	0.00311155	0.00313657
indoor lighting	0.00367905	0.00371899	0.00374535	0.0037538	0.00378158
far-away horizon	0.00531631	0.005401	0.00546001	0.00560656	0.00575712
clouds	0.00646776	0.00654812	0.00665955	0.0066942	0.00684807

Table S6. Environmental indicators of aesthetic landscape quality.

**Visual concept**	Theory	Indicator(s)	Description	Dataset	Source
Naturalness	Important visual factor related to nature's restorative effects on mental health[1,2] and the innate biological need to affiliate with nature[3]. The aesthetic value of water, in particular, is linked to its perceived naturalness[4–6].	% ecosystem type	The % of ecosystem types per grid cell	EEA ecosystem type map at $100 \times 100m$	7
Visual scale	A key driver of peoples' aesthetic experience of the landscape. Landscapes that are both open and offer refuge are said to be more attractive because of our evolutionary history as both predator and prey[8]. Thus, higher elevation areas and differences in the landscape are likely to offer a greater aesthetic appeal.	Relief (m)	The difference in elevation within each grid cell	EU DEM at $25 \times 25m$	9
Complexity	According to Kaplan's Informational Processing Theory, complexity satisfies our physiological need to explore, providing content and things to think about[4]. Complexity in the landscape can manifest itself in terms of the number and distribution of landscape attributes. Indicators to represent landscape complexity therefore frequently draw upon landscape metrics in landscape ecology such as the PDI and SDI[10].	Patch Density Index (PDI)	Fragmentation, number of distinct ecosystem patches	EEA ecosystem type map at $100 \times 100m$	7
		Shannon Diversity Index (SDI)	Number of ecosystems and their spatial proportion	EEA ecosystem type map at $100 \times 100m$	7
Uniqueness	Takes into account the distinctiveness of an area[11]. For example, a natural feature can hold a much larger aesthetic value in the urban environment than in a more natural context. Uniqueness is also related to concepts of imageability and historicity which recognise the impression landscape elements with a cultural significance can have on the viewer[10,12].	% ecosystem type versus surrounding area	Relative difference in the percentage area of ecosystem types in grid cells within $10km$	EEA ecosystem type map at $100 \times 100m$	7
		Historic POI	Number of historic points of interest per grid cell[1]	OSM	13

[1]*The OSM API was used to calculate historical point features per grid cell. This included archaeological sites, ruins, castles and churches (a full list can be found in Table S10). All spatial features returned by the API query that were not points were converted to point features by calculating their centroids.*

Table S7. SoN ResNet-50 test statistics. Model accuracy is reported using root mean squared error (RMSE) and Kendall's τ,a ranking correlation coefficient.

**Epoch**	**RMSE**	**Kendall's τ**
1	0.6998	0.6299
2	0.7008	0.6339
3	0.7079	0.6381
4	0.6909	0.6361
5	0.7546	0.6368
6	0.6926	0.6375
7	0.6997	0.6328
8	0.7491	0.6275
9	0.7189	0.6215
10	0.7325	0.6172

Table S8. Most correlated indicators. "(s)" denotes an ecosystem in surrounding area variable.

Indicator	2nd indicator	Pearson's R
I1 farmland	I1 farmland (s)	0.904
J2 low density buildings (s)	I2 gardens and parks (s)	0.887
J1 cities, towns and villages (s)	I2 gardens and parks (s)	0.886
G3 coniferous woodland (s)	G5 lines of trees (s)	0.858
E2 mesic grasslands	E2 mesic grasslands (s)	0.819
F2 alpine shrub	F2 alpine shrub (s)	0.816
I2 gardens and parks (s)	J4 roads (s)	0.802
J1 cities, towns and villages (s)	J4 roads (s)	0.793
D1 bogs	D1 bogs (s)	0.777
J1 cities, towns and villages (s)	J2 low density buildings (s)	0.760
J2 low density buildings (s)	J4 roads (s)	0.752
F2 alpine shrub (s)	E4 alpine grasslands (s)	0.748
G1 deciduous woodland	G1 deciduous woodland (s)	0.746
J2 low density buildings	J2 low density buildings (s)	0.744
I2 gardens and parks	I2 gardens and parks (s)	0.713
G3 coniferous woodland	G3 coniferous woodland (s)	0.713
J1 cities, towns and villages	I2 gardens and parks	0.704

Table S9. Time-series analysis - additional data sources.

Dataset	Source
National Parks (England)	https://naturalengland-defra.opendata.arcgis.com/
Cairngorms National Park Designated Boundary	https://spatialdata.gov.scot/
Loch Lomond and The Trossachs National Park Designated Boundary	https://spatialdata.gov.scot/
National Parks (Wales)	https://lle.gov.wales/
MOD10CM.V6	https://nsidc.org/[14]

Table S10. OSM historic POI.

**Category**	**Description**
aqueduct	A historic structure to convey water
archaeological site	A place in which evidence of past activity is preserved.
building	Unspecified historic building.
castle	Used for various kinds of castles, palaces, fortresses, manors, stately homes, kremlins, shiros and other.
castle wall	A fortification surrounding the bailey of a castle
church	A building with historical value for Christian religious activities, particularly for worship services.
city gate	A city gate within a city wall
citywalls	A citywall is a fortification used to defend a city.
farm	A historical farm, kept in its original state.
fort	A military fort, a stand-alone defensive structure which differs from a castle in that there is no permanent residence
manor	Historic manors/mansions having different use today \\
monastery	Building/place that was a monastery.
monument	A memorial object, which is especially large, built to remember, show respect to a person or group of people or to commemorate an event.
ruins	Remains of structures that were once complete, but have fallen into partial or complete disrepair.
rune stone	Stones, boulders or bedrock with historical runic inscriptions.
tower	This property distinguishes a tower as historic
wayside cross	A historical cross, symbol of christian faith.
wayside shrine	A shrine often showing a religious depiction. Also for modern shrines.

SI References

1. Ulrich, R. S. Visual landscapes and psychological well-being. *Landsc. Res.* **4**, 17–23 (1979).
2. Ulrich, R. S. View through a Window May Influence Recovery from Surgery. *Science (80-.).* **224**, 420–421 (1984).
3. Kellert, S. R. & Wilson, E. O. *The Biophilia Hypothesis*. (Island Press/Shearwater Books, 1993).
4. Kaplan, R. & Kaplan, S. *The Experience of Nature*. (Cambridge University Press, 1989).
5. Nasar, J. L. The evaluative image of places. in *Person–environment psychology: New directions and perspectives, 2nd ed.* 117–168 (Lawrence Erlbaum Associates Publishers, 2000).
6. Nasar, J. L. & Li, M. Landscape mirror: the attractiveness of reflecting water. *Landsc. Urban Plan.* **66**, 233–238 (2004).
7. EEA. Ecosystem types of Europe. (2019). Available at: https://www.eea.europa.eu/data-and-maps/data/ecosystem-types-of-europe-1.
8. Appleton, J. *The Experience of Landscape*. (John Wiley and Sons, 1975).
9. EEA. Copernicus Land Monitoring Service - EU-DEM. (2017). Available at: https://www.eea.europa.eu/data-and-maps/data/copernicus-land-monitoring-service-eu-dem.
10. Ode, Å., Tveit, M. S. & Fry, G. Capturing Landscape Visual Character Using Indicators: Touching Base with Landscape Aesthetic Theory. *Landsc. Res.* **33**, 89–117 (2008).
11. Jessel, B. Elements, characteristics and character – Information functions of landscapes in terms of indicators. *Ecol. Indic.* **6**, 153–167 (2006).
12. Tveit, M., Ode, Å. & Fry, G. Key concepts in a framework for analysing visual landscape character. *Landsc. Res.* **31**, 229–255 (2006).
13. Padgham, M., Rudis, B., Lovelace, R. & Salmon, M. osmdata. *J. Open Source Softw.* **2**, (2017).
14. Hall, D. K. & Riggs, G. A. MODIS/Terra Snow Cover Monthly L3 Global 0.05Deg CMG, Version 6. *NASA National Snow and Ice Data Center Distributed Active Archive Center* (2015). doi:https://doi.org/10.5067/MODIS/MOD10CM.006
